# A REINFORCEMENT LEARNING APPROACH TO ENHANCE PHYSICAL ACTIVITY AND REDUCE FALL RISK IN OLDER ADULTS

**DOI:** 10.1093/geroni/igae098.3473

**Published:** 2024-12-31

**Authors:** Chang Liu, Ladda Thiamwong, Tho Nguyen, Jethro Raphael Suarez, Joon-Hyuk Park, Qian Lou, Rui Xie

**Affiliations:** University of Central Florida, Orlando, Florida, United States; University of Central Florida, Orlando, Florida, United States; University of Central Florida, Orlando, Florida, United States; University of Central Florida; University of Central Florida, Orlando, Florida, United States; University of Central Florida, Orlando, Florida, United States; University of Central Florida, Orlando, Florida, United States

## Abstract

The proliferation of sensing technology enables us to accurately track physical activity (PA) using wearable devices. Consequently, based on the PA patterns provided, it offers us an opportunity to design personalized interventions delivered via notifications, aiming to promote healthy behaviors and eventually reduce fall risk among older adults. We assessed minute-level accelerometer-based PA data, in total 1.24 million observations, from 134 participants (average age 74.26, SD 6.85, female 88.8% and 70.15% have no history of falls) in our Physio-Feedback Exercise Program (PEER) and control groups. The study targets the reduction of fall risk and the improvement of PA levels in economically disadvantaged older adults recruited in 2023. We employed a reinforcement learning (RL) approach with a policy network that continuously learns steps and vector magnitude from participants, aiming to find an optimal policy to encourage participants to take the action of ‘standing’. We assigned a positive reward when participants achieved Moderate to Vigorous Physical Activity status, a neutral reward for Light Physical Activity, and a negative reward for sedentary behavior. A three-layer fully connected neural network with sigmoid activation functions identified a significant difference in action probabilities, with the control group averaging 0.6456 and the PEER group at 0.7192. Significantly, from 9 AM to 6 PM, probabilities surged for both groups, peaking at 0.7438 (control) and 0.8188 (PEER). After 6 PM, probabilities declined across all groups. The probability of an event is directly proportional to its likelihood; therefore, a higher probability corresponds to the greater likelihood of the event occurs.

